# S129. DOES TREATMENT RESISTANT SCHIZOPHRENIA PRESENT A CHARACTERISTIC SYMPTOMATIC SIGNATURE?

**DOI:** 10.1093/schbul/sby018.916

**Published:** 2018-04-01

**Authors:** Bruno Bertolucci, José Cássio Pitta, Cinthia Higuchi, Cristiano Noto, Deyvis Rocha, Dan Joyce, Christoph Correll, Rodrigo Bressan, Ary Gadelha

**Affiliations:** 1 UNIFESP; 2 Institute of Psychiatry, King’s College; 3 The Zucker Hillside Hospital, Hofstra North Shore LIJ School of Medicine

## Abstract

**Background:**

Treatment-resistant schizophrenia (TRS) may underlie a specific biological signature among patients with schizophrenia. The main lines of evidence suggest a glutamatergic rather than dopaminergic dysfunction in TRS, with lower levels of striatal dopamine and higher levels of glutamate in anterior cingulate. Whether this biological signature relates to a distinct symptomatic profile remains unclear. Our objective is to define a symptom profile of patients with TRS.

**Methods:**

We used two samples of patients with schizophrenia. First, we followed a discovery sample of inpatients (n=203) to prospectively identify TRS predictors, then we tested the predictors in a replication sample of outpatients (n=207). The samples were collected independently. All patients were assessed with the Positive and Negative Syndrome Scale (PANSS), the Clinical Global Impressions-Severity Scale (CGI-S) and the Global Assessment of Functioning Scale (GAF). Diagnosis was confirmed using the Structured Clinical Interview for DSM-IV Axis I Disorders (SCID-I). TRS was defined according the criteria of the Schizophrenia Algorithm of the International Psychopharmacology Algorithm Project (IPAP). Initially, we tested if patients with disorganized subtype were more likely to be TRS, and grouped the patients into disorganized or non-disorganized schizophrenia according to SCID-I. Then, we checked which PANSS items at the baseline predicted TRS at the follow-up through multiple logistic regression analyses. A receiver operating characteristic (ROC) curve with the best items was performed at the follow-up.

**Results:**

TRS was more common in disorganized schizophrenia in the inpatient sample (73.8% vs 22.4%, P < 0.001) and in the outpatient sample (68.2% vs 28.2%, P < 0.001) in comparison to non-disorganized schizophrenia. They also presented worse scores on PANSS, CGI-S and GAF (P < 0.001). In the second step, three PANSS items, P2 (conceptual disorganization), N5 (difficulty in abstract thinking) and G9 (unusual thought content), predicted TRS with 78.4% accuracy (P = 0.011, P = 0.010 and P <0.001). The ROC analysis using the sum of PE+N5G+G9 predicted TRS with a sensitivity of 72.3%, and a specificity of 82.4%. In the outpatient sample, logistic regression analysis of the model P2+N5+G9 discriminated TRS with 69.3% accuracy (P <0.001).

**Discussion:**

Non-paranoid clinical presentations, specially disorganized characteristics, may consist in clinical markers of TRS. Further Cross-validation of such clinical findings and biological features may improve prediction of TRS

